# Influence of initial dose intensity on efficacy of FOLFIRINOX in patients with advanced pancreatic cancer

**DOI:** 10.18632/oncotarget.26633

**Published:** 2019-03-05

**Authors:** Satoshi Kobayashi, Makoto Ueno, Katsuhiro Omae, Hidekazu Kuramochi, Masato Terao, Nobumasa Mizuno, Masato Ozaka, Hideki Ueno, Kazuhiro Uesugi, Noritoshi Kobayashi, Marina Kobayashi, Akiko Todaka, Akira Fukutomi

**Affiliations:** ^1^ Department of Gastroenterology, Hepatobiliary and Pancreatic Medical Oncology Division, Kanagawa Cancer Center, Yokohama, 241-0815, Japan; ^2^ Clinical Research Center, Clinical Research Promotion Unit, Shizuoka Cancer Center, Shuntogun, 411-8777, Japan; ^3^ Department of Medical Oncology, Tokyo Women’s Medical University Yachiyo Medical Center, Yachiyo, 276-8524, Japan; ^4^ Department of Medical Oncology, Fukuyama City Hospital, Fukuyama, 721-8511, Japan; ^5^ Department of Gastroenterology, Aichi Cancer Center Hospital, Nagoya, 464-8681, Japan; ^6^ Department of Gastroenterology, The Cancer Institute Hospital of Japanese Foundation for Cancer Research, Tokyo, 135-8550, Japan; ^7^ Department of Hepatobiliary and Pancreatic Oncology, National Cancer Center Hospital, Tokyo, 104-0045, Japan; ^8^ Department of Gastroenterology, National Hospital Organization Shikoku Cancer Center, Matsuyama, 791-0280, Japan; ^9^ Department of Oncology, Yokohama City University, Yokohama, 236-0004, Japan; ^10^ Clinical Trial Promotion Section, Shizuoka Industrial Foundation Pharma Valley Center, Shuntogun, 411-0934, Japan; ^11^ Department of Gastrointestinal Oncology, Shizuoka Cancer Center, Shuntogun, 411-8777, Japan

**Keywords:** irinotecan, fluorouracil, oxaliplatin, leucovorin, dose response relationship

## Abstract

The combination of fluorouracil, leucovorin, irinotecan, and oxaliplatin (FOLFIRINOX) is the standard of care for advanced pancreatic cancer, but causes hematological and gastrointestinal toxicities, leading to treatment delay and dose reduction; optimal modification based on toxicities is needed. Therefore, we evaluated the effect of initial relative dose intensity (RDI) on FOLFIRINOX efficacy by conducting a Japanese nationwide survey. We evaluated overall survival (OS) and progression-free survival (PFS) of patients administered two or more cycles of FOLFIRINOX, and determined RDIs for each drug within the first two cycles. RDI’s effect on efficacy was evaluated using a multivariate analysis with a Cox regression hazard model. Of 399 patients enrolled, 359 and 346 were evaluated for OS and PFS, respectively. Median RDI was 71.8%, 64.7%, 23.4%, and 76.9% for oxaliplatin, irinotecan, and bolus and continuous infusions of 5-FU, respectively. A high RDI for 5-FU bolus resulted in poor prognosis in terms of PFS (hazard ratio: 1.34; *p* = 0.022) and negatively correlated with objective response (coefficient: −0.70; *p* = 0.021), and a high RDI for CPT-11 positively correlated with objective response (coefficient: 1.02; *p* = 0.031). In conclusion, low and high RDIs for irinotecan and 5-FU bolus, respectively, resulted in poor FOLFIRINOX efficacy.

## INTRODUCTION

Pancreatic adenocarcinoma is the fourth leading cause of death in the US and accounted for approximately 40,000 deaths in 2014 [1]. It was also the fourth leading cause of death in Japan in 2013 [2]. The disease can only be cured by surgical resection; however, it is often detected only in the unresectable stage. Thus, various systemic chemotherapies have been evaluated for the management of advanced pancreatic adenocarcinoma. The combination of 5-fluorouracil (5-FU), leucovorin (*l*-LV), irinotecan (CPT-11), and oxaliplatin (L-OHP), FOLFIRINOX, has been shown to improve overall survival (OS) in patients with metastatic pancreatic adenocarcinoma compared with gemcitabine treatment [3]. Thus, FOLFIRINOX is now the standard of care in the treatment guideline for metastatic pancreatic adenocarcinoma [4].

FOLFIRINOX can cause hematological and non-hematological toxicities, which are associated with treatment delay, dose reduction, or both in subsequent cycles and decreased dose intensity (DI). Thus, a reduction in the initial dose is warranted, and some studies have reported that the efficacy of a modified regimen was non-inferior to that of the original [5–8]. Based on these results, a modified FOLFIRINOX regimen is widely used globally [6, 7, 9]. However, the initial doses or post-initiation dose modifications were different in each study, and the optimal modification remains unknown. Therefore, in this study, we evaluated the effects of relative DI (RDI) of each constituent of FOLFIRINOX on survival benefits in patients with advanced pancreatic cancer, to clarify the optimal initiation and ongoing treatment dose modifications.

## RESULTS

### Patients

Of the 406 patients, seven were excluded for the following reasons: treatment did not coincide with the study period, double registration (two patients each), treatment did not include CPT-11, disease status was resectable, and voluntary withdrawal (one patient each). Moreover, we excluded an additional 40 patients because they discontinued FOLFIRINOX in the first cycle and 53 in the analysis of progression-free survival (PFS) because they showed progression in the second cycle. Thus, 359 and 346 patients were enrolled in the background and OS analysis and PFS analysis, respectively. Patients’ characteristics are shown in Table 1.

**Table 1:** Patient characteristics

Factors	*n* (%)
Sex	
Female Male	115 (32.0) 244 (68.0)
Age	
<65 years ≥65 years	223 (62.1) 136 (37.9)
Disease status	
Locally advanced Metastatic Recurrence	73 (20.3) 212 (59.1) 74 (20.6)
History of prior chemotherapy	
<No Yes	265 (73.8) 94 (26.2)
ECOG Performance status	
0 1, 2	257 (71.6) 102 (28.4)
UGT1A1	
Wild Single Double	201 (56.0) 134 (37.3) 15 (4.2)
CA19-9, U/mL median (range)	1071.0 (0.4–368500)
Albumin, g/dL median (range)	3.9 (2.3–5.3)
CRP, mg/dL median (range)	0.3 (0.0–12.4)

Abbreviations: ECOG, Eastern Cooperative Oncology Group; CA19-9, carbohydrate antigen 19-9; CRP, C-reactive protein.

### Treatment course

The original and modified FOLFIRINOX regimens were administered to 120 and 239 patients, respectively. G-CSF was used until the end of the second cycle in 99 patients (27.6%), although it was used prophylactically in the first cycle only in one patient. In the second cycle, treatment was delayed in 267 patients (74%), and the doses of L-OHP, CPT-11, 5-FU bolus, and 5-FU ci were decreased in 146 (41%), 304 (85%), 273 (76%), and 88 (24%) patients, respectively. The reasons for treatment delay were neutropenia, leucopenia, anorexia, febrile neutropenia, thrombocytopenia, and diarrhea in 171, 46, 13, 12, eight, and seven patients, respectively. The reasons for dose reduction were neutropenia, leucopenia, anorexia, nausea, diarrhea, febrile neutropenia, fatigue, and thrombocytopenia in 151, 32, 30, 15, 14, 12, 7, and 7 patients, respectively (there was some overlap).

**Figure 1:**
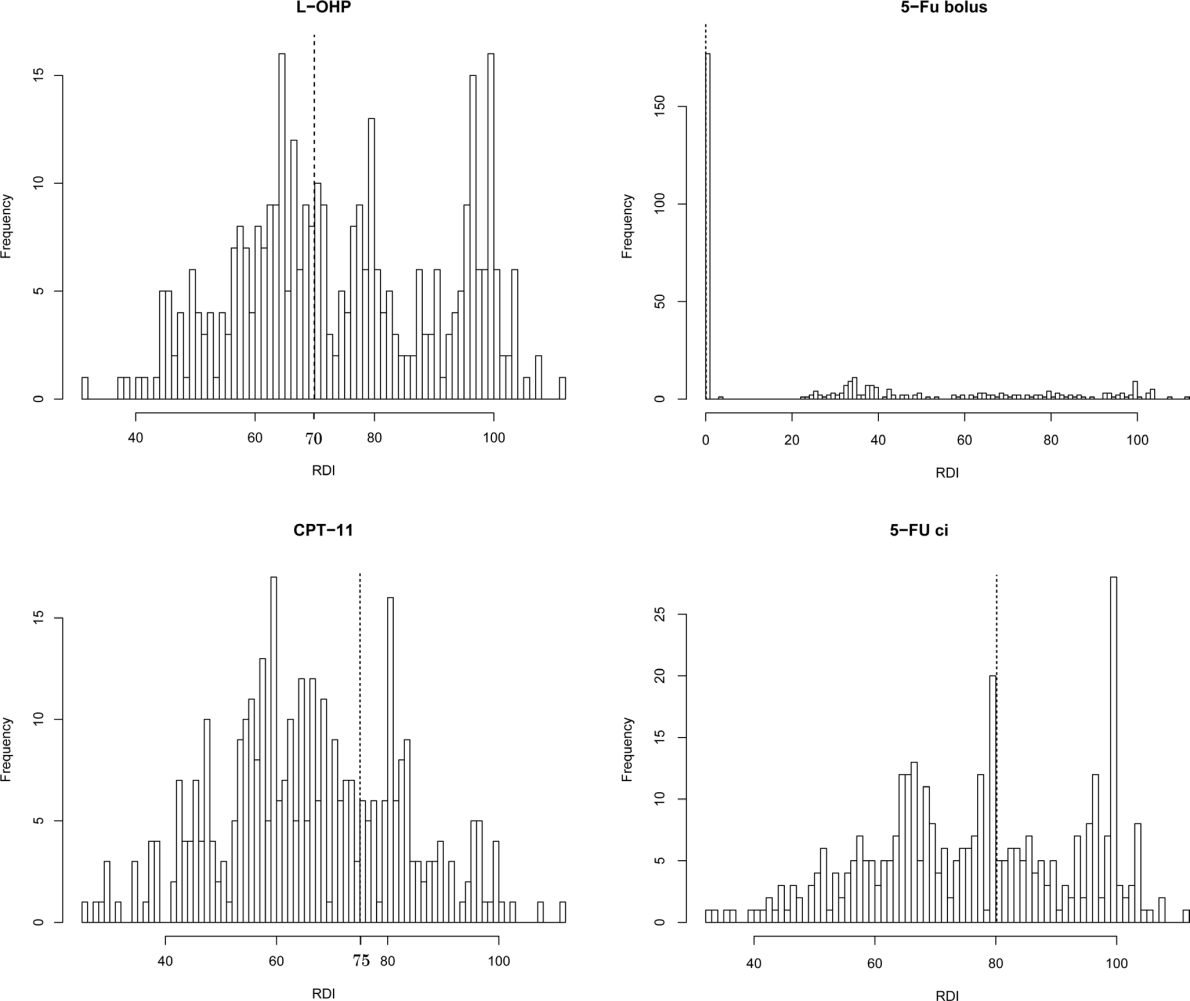
Histogram of the relative dose intensities (RDIs) of each agent in FOLFIRINOX within the first two cycles The dotted line shows the cut-off values for each agent.

The distribution of the initial RDIs for each agent is shown in Figure 1. The median RDIs for L-OHP, CPT-1, 5-FU bolus, and 5-FU ci in the first two cycles were 71.8%, 64.7%, 23.4%, and 76.9 %, respectively. Using the cut-off value described in the Methods section, 197 (55%), 107 (30%), 179 (50%), and 144 (40%) patients administered L-OHP, CPT-11, 5-FU bolus, and 5-FU ci, respectively, were considered the high RDI group. There was a strong correlation in the RDIs between L-OHP and CPT-11, and L-OHP and 5-FU ci, and moderate correlation between CPT-11 and 5-FU ci (Supplementary Figure 1). The factors affecting the RDIs of each agent are shown in Table 2. Patients who were started on the original dose of CPT-11 tended to have a high RDI for CPT-11 (odds ratio: 2.04, 95% CI: 1.25–3.32, *p*-value = 0.004). Using the 5-FU bolus was related to a high RDI of the 5-FU bolus.

**Table 2:** Effects of patient characteristics on the relative dose intensities (RDIs) of each agent

	L-OHP ≥ 70%		CPT-11 ≥ 75%		5-FU bolus > 0%		5-FU ci. ≥ 80%	
Factors (Reference) levels	Odds Ratio (95% CI)	*p*	Odds Ratio (95% CI)	*p*	Odds Ratio (95% CI)	*p*	Odds Ratio (95% CI)	*p*
Sex (Male) Female			2.36 (1.35, 4.26)	<0.005			1.53 (0.93, 2.54)	0.094
Age (<65) ≧65								
Disease status (LA) Metastatic Recurrence	2.00 (1.14, 3.52) 1.20 (0.61, 2.36)	<0.05 0.600					1.67 (0.93–3.05) 0.99 (0.48–2.03)	0.090 0.972
History of PT (No) Yes								
ECOG PS (0) 1/2							0.43 (0.24, 0.73)	<0.005
UGT1A1(Wild) Single hetero Double hetero								
Albumin, g/dl (<3.5) ≧3.5	2.02 (1.11, 3.76)	<0.05	2.20 (1.10, 4.71)	<0.05			2.53 (1,32, 5.07)	<0.01
CRP, mg/dl (<2.0) ≧2.0	3.25 (1.46, 7.92)	<0.05	2.85 (1.36, 6.13)	<0.01			2.67 (1.23, 5.98)	<0.05
ID of L-OHP (Orig.) Decreased								
ID of CPT-11 (Orig.) Decreased			0.30 (0.14, 0.62)	<0.005				
ID of 5-Fu bolus (Orig.) Decreased	0.26 (0.06, 0.91)	<0.05	2.02 (1.01, 4.30)	0.056	8.41 × 10^−24^ (0.00–0.00)	0.999	1.44 (0.91, 2.27)	0.121
ID of 5-Fu ci. (Orig.) Decreased	1.42 (0.91, 2.22)	0.121					8.82 × 10^−8^ (NA-8.07 × 10^−24^)	0. 981

Abbreviations: L-OHP, oxaliplatin; CPT-11, irinotecan; 5-FU, fluorouracil; ci, continuous infusion; CI, confidence interval; LA, locally advanced; PT, prior therapy; ECOG PS, Eastern Cooperative Oncology Group Performance status; CRP, C-reactive protein; ID, initial dose; Orig, original dose; NA, not applicable.

### Efficacy

The median OS was 11.3 months (95% CI: 10.1–12.6). The results of a univariate analysis of OS according to the initial RDIs of each agent are shown in Supplementary Figure 2. Patients who received 5-FU bolus at a high RDI had a poor prognosis (hazard ratio: 1.32 [95% CI: 1.00–1.71], *p* = 0.042), whereas the RDIs of the other three agents were not significant factors; the hazard ratios were 1.12 (95% CI: 0.86–1.46, *p* = 0.21), 0.83 (95% CI: 0.62–1.12, *p* = 0.11), and 0.81 (95% CI: 0.62–1.06, *p* = 0.12) for L-OHP, CPT-11, and 5-FU ci, respectively. A multivariate analysis showed that the RDIs of all four agents were not significant factors affecting OS while recurrent disease (vs. local), a history of chemotherapy, ECOG PS of 1–2 (vs. 0), serum level of carbohydrate antigen 19-9 ≥ 2000 U/mL, and C-reactive protein level ≥ 2.0 mg/dL were poor prognostic factors for OS (Supplementary Table 1).

**Figure 2:**
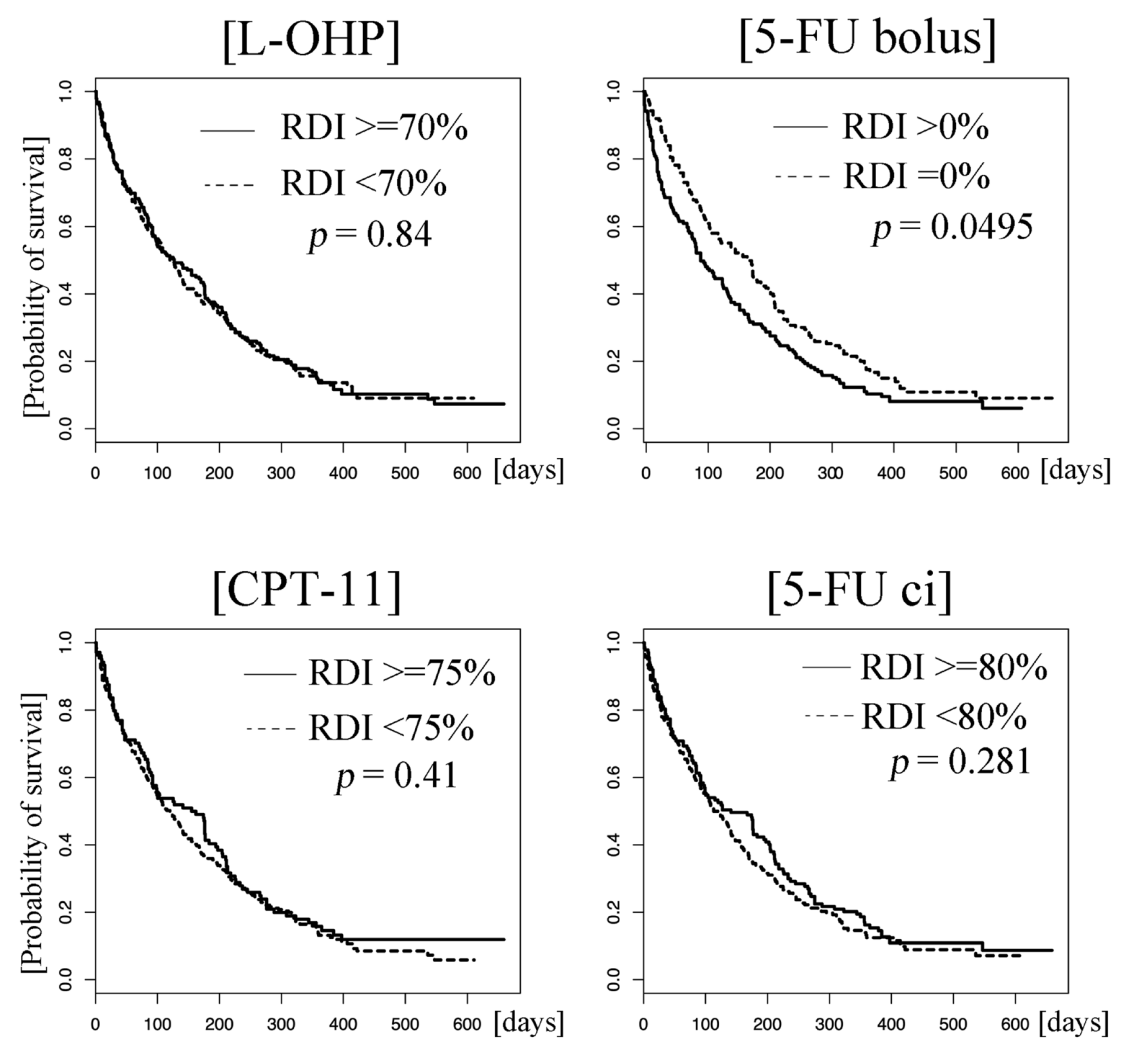
Kaplan–Meier curves of progression-free survival (PFS) Patients who received the drugs at higher and lower relative dose intensities (RDIs) than the cut-off values are represented by solid and dotted lines, respectively. There was a statistical difference in progression-free survival between patients who received 5-FU bolus at higher and lower relative dose intensities (> 0% vs. 0%, *p* = 0.0050). No such differences were observed for L-OHP (≥ 70% vs. < 70%), CPT-11 (≥ 75% vs. < 75%), and 5-FU ci (≥ 80% vs. < 80%) (*p* = 0.84, 0.41, and 0.28, respectively). Abbreviations: L-OHP, oxaliplatin; CPT-11, irinotecan; ci, 5-FU, fluorouracil; continuous infusion.

The median PFS was 4.2 months (95% CI: 3.7–4.8). Univariate analysis showed that patients who received 5-FU bolus at a high RDI had a poor prognosis (hazard ratio: 1.38 [95% CI: 1.10–1.73], *p* = 0.0050). The RDIs of the other three agents were not significant factors; the hazard ratios were 0.98 (95% CI: 0.78–1.22, *p* = 0.85), 1.09 (95% CI: 0.86–1.39, *p* = 0.47), and 0.97 (95% CI: 0.78–1.21, *p* = 0.77) for L-OHP, CPT-11, and 5-FU ci, respectively (Figure 2). Multivariate analysis also demonstrated that the higher RDI for 5-FU bolus was a poor prognostic factor for PFS: the hazard ratio was 1.34 (95% CI: 1.04–1.72) with a *p*-value of 0.022 (Table 3).

**Table 3:** Prognostic factors for progression-free survival (PFS)

Variable	Hazard ratio	95% CI	*p*-value
L-OHP ≥ 70%	1.13	0.80–1.61	0.49
CPT-11 ≥ 75%	0.89	0.61–1.31	0.56
5-FU bolus > 0%	1.34	1.04–1.72	0.022
5-FU ci. ≥ 80%	0.90	0.59–1.36	0.60
Sex	0.99	0.76–1.28	0.93
Age	0.86	0.66–1.11	0.24
Disease status (vs. local)			
Metastatic	1.29	0.95–1.77	0.11
Recurrence	1.51	1.02–2.22	0.037
History of therapy	1.39	1.05–1.84	0.021
ECOG PS	1.41	1.07–1.87	0.015
G-CSF use	1.00	0.74–1.36	0.99
UGT1A1 (Ref: Wild)			
Single variant	0.98	0.77–1.26	0.89
Double variant	1.16	0.63–2.14	0.63
CA19-9^*^	1.34	1.04–1.73	0.022
Albumin^*^	1.00	0.73–1.36	0.99
CRP^*^	1.80	1.22–2.66	0.003

^*^ continuous value.

Abbreviations: L-OHP, oxaliplatin; CPT-11, irinotecan, 5-FU, fluorouracil; ci, continuous infusion; ECOG PS, Eastern Cooperative Oncology Group Performance status; G-CSF, granulocyte-colony stimulating factor; CA19-9, carbohydrate antigen 19-9; CRP, C-reactive protein.

Partial response (PR) was observed in 72 patients, which resulted in the objective response rate being 20.0%. The results of multivariate analysis for the objective response are shown in Table 4; the high RDI for CPT-11 and low RDI for 5-FU bolus (vs. >0%) were significant factors affecting objective response. The coefficient values were 1.02 (95% CI: 0.12–1.98, *p* = 0.031) and 0.70 (95% CI: **−**1.30 – **−**0.11, *p* = 0.021).

**Table 4:** Multivariate analysis of objective response

Variable	Coefficient	*p*-value
L-OHP ≥ 70%	−0.39	0.39
CPT-11 ≥ 75%	1.02	0.031
5-FU (bolus) > 0%	−0.70	0.021
5-FU (duration) ≥ 80%	−0.083	0.87
Sex: Female	−0.60	0.050
Age ≥ 65 years	−0.12	0.70
Disease status (vs. local)		
Metastatic	0.46	0.25
Recurrent	−0.13	0.79
History of therapy	−0.16	0.65
ECOG PS	−0.51	0.13
G-CSF use	0.53	0.14
UGT1A1 (Ref: Wild)		
Single variant	0.14	0.63
Double variant	−0.55	0.51
CA19-9^*^	−0.22	0.46
Albumin^*^	−0.28	0.45
CRP^*^	0.33	0.46

^*^continuous value.

Abbreviations: L-OHP, oxaliplatin; CPT-11, irinotecan, 5-FU, fluorouracil; ci, continuous infusion; ECOG PS, Eastern Cooperative Oncology Group Performance status; G-CSF, granulocyte-colony stimulating factor; CA19-9, carbohydrate antigen 19-9; CRP, C-reactive protein.

## DISCUSSION

We compared the efficacy of FOLFIRINOX in patients with advanced pancreatic cancer who received drugs at high and low RDIs in the first two cycles to evaluate the impact of RDI on treatment efficacy. The initial RDIs for 5-FU bolus had a negative correlation with PFS and objective response, whereas the RDIs for CPT-11 had a positive effect on objective response.

A modified regimen in which the initial dose is decreased is widely used as it is safe. In addition, a modified regimen might have comparable efficacy to that of the original one, although this hypothesis has not been confirmed in a prospective randomized trial [6, 7]. However, the results of the current study support the maintenance of a high RDI for CPT-11 and non-reduction of the initial dose of CPT-11 to achieve an objective response. Only 59 of 237 patients (24.9%) for whom the initial CPT-11 dose was reduced could receive the second cycle with neither treatment delay nor additional dose reduction. Furthermore, the initial dose of CPT-11 did not affect the RDIs for L-OHP, 5-FU bolus, and 5-FU ci (Table 2), and the original CPT-11 dose was acceptable in terms of the RDIs for the other three agents. Taking this into account, non-reduction of the dose of CPT-11 at the first and second cycle would be favorable, especially in patients with locally advanced or borderline resectable pancreatic cancer, as the aim of the treatment is tumor shrinkage for the following surgery. Regarding the 5-FU bolus, the administration at initiation led to a high RDI and had a negative effect on the PFS and objective response. We speculated that it could be explained by the results of this study that initiation with 5-FU bolus decreased the RDI of CPT-11 (Table 2). Another possible reason was the low RDI of 5-FU ci in patients who received 5-FU bolus although the decrease was not statistically significant. The meta-analysis which showed the use of 5-FU ci was superior to that of 5-FU bolus in colorectal cancer [10] might support our speculation, although 5-FU ci did not show statistically significant effects with respect to any indicators of efficacy in our study and the type of cancer was different. In addition, patient’s characteristics had an influence because there were more patients who had prior history of chemotherapy in the high RDI group of 5-FU bolus than in the low RDI group (Supplementary Table 2). Nonetheless, optimal dose modification remains a clinical question in patients with metastatic pancreatic cancer whose primary treatment endpoint is OS, as none of the RDIs significantly influence OS.

The efficacy of systemic chemotherapy using cytotoxic agents for unresectable solid tumors basically depends on the dose rather than DI [11, 12]; however, in some types of cancer such as lung, breast, and ovarian cancers, the RDI for chemotherapy with cytotoxic agents was related to its efficacy [11, 13–15]. Regarding FOLFIRINOX for advanced pancreatic cancer, the effect of RDI on the efficacy has not been documented, except for in a study by Lee *et al.* [16] who reported that the cumulative RDI of FOLFIRINOX >70% was related to the radiological response. We evaluated the initial RDI, not the cumulative value, to avoid potential biases; a decrease in the dose of each agent due to toxicities, especially cumulative neuropathy caused by L-OHP, is often required at a later stage and may result in the cumulative RDI being lower in patients who have longer PFS than in those who discontinue FOLFIRINOX within a short period. In addition, we separated the RDIs of each agent to clarify doses that should not be reduced. Another concern is the cut-off value for evaluating the influence of RDI on efficacy. Although we set the cut-off values based on a model case, they were almost the same as the median RDIs in the phase III study of FOLFIRINOX: 78%, 81%, and 82% for L-OHP, CPT-11, and 5-FU, respectively [3]. In other types of cancer, RDI cut-off values ranging from 70% to 80% were reported as a predictive factor for prolonged PFS [13–15], and we consider that the cut-off values in the current study are acceptable.

There are some limitations to this study. First, the patients’ characteristics were diverse. Disease status (locally advanced, metastatic, or recurrent) and the indication of FOLFIRINOX (first-line or later) were obviously important for evaluating the prognostic factors for OS. Additionally, because of the variations in patient characteristics, the RDIs of none of the agents had a significant influence on OS. Second, the study design was retrospective, and decisions of treatment delay and dose reduction were dependent on the physician’s discretion. Third, the study excluded patients who discontinued the regimen within two cycles to evaluate the influence of initial RDIs on the following prognosis. Therefore, the results of our study cannot predict the probability of early progression within two cycles. Fourth, we did not evaluate the prophylactic use of G-CSF. G-CSF can lead to a higher RDI without a decrease in the initial dose, although treatment delay, dose reduction, or both are not only attributable to neutropenia but also to thrombocytopenia or non-hematologic toxicities.

Despite these limitations, to the best of our knowledge, this is the first study to evaluate the effect of the initial RDI of each drug constituent of FOLFIRINOX on its efficacy in patients with advanced pancreatic cancer. CPT-11 RDI of >75% predicts the radiological response and 5-FU RDI of >0% predicts poor efficacy of FOLFIRINOX. Therefore, initiation of CPT-11 administration at the original dose with omission of 5-FU bolus and dose reductions for agents other than CPT-11 in cases of adverse events might be the best approach to achieve efficacy. A prospective study is needed to verify the findings.

## MATERIALS AND METHODS

### Patients

In a nationwide, multicenter, observational study of FOLFIRINOX chemotherapy (JASPAC06), each participating institution consecutively registered all patients with unresectable or recurrent pancreatic cancer who received FOLFIRINOX therapy at least once within 1 year from December 20, 2013 (the approval date of L-OHP and CPT-11 for advanced pancreatic cancer in Japan). In total, 406 patients from 27 institutions were registered between November 2014 and May 2015.

Written informed consent was obtained from patients who had been receiving FOLFIRINOX at the time of registration or those who initiated FOLFIRINOX during the registration period. Patients who had discontinued FOLFIRINOX until the time of registration were informed of their right to opt out via public announcements at each participating institution. This study was approved by the institutional review board of each participating institution and conducted based on the Ethical Guidelines of Epidemiological Research. This study was registered on the University Hospital Medical Information Network (UMIN000014658).

### Treatment

The original regimen consisted of 85 mg/m^2^ L-OHP, 200 mg/m^2^ *l*-LV, 180 mg/m^2^ CPT-11, and 400 mg/m^2^ 5-FU (5-FU bolus), followed by a continuous intravenous infusion of 2400 mg/m^2^ 5-FU (5-FU ci) for 46 h every 2 weeks, which was the same as that in previous prospective studies [3, 17]. The dose of each drug at baseline and during treatment was sometimes reduced, and treatment was delayed at the physician’s discretion based on the patient’s condition and toxicities observed (Supplementary Table 3).

### Assessment

The data cut-off time was December 2015. DI was defined as the delivered dose of each individual agent divided by the planned dose of the original FOLFIRINOX regimen, which were calculated using body surface area measured using DuBois formula. The RDI was defined as the ratio of actual DI to the DI designed per specific period, similar to that in the Hryniuk model [18]. We used the RDI of the first two cycles for the following evaluation. OS was defined as the duration from the initiation date of the third cycle to death from any cause. PFS was defined as the duration from the initiation date of the third cycle to the date of documented disease progression or death from any cause. Objective responses were evaluated using computed tomography or magnetic resonance imaging every 2 or 3 months at the physician’s discretion (not specified in the protocol) in accordance with the Response Evaluation Criteria in Solid Tumors, version 1.1 [19].

### Statistics

If the RDIs of L-OHP, CPT-11, 5-FU bolus, and 5-FU ci. were higher than the cut-off values of 70%, 75%, 0%, and 80%, respectively, the level was defined as high; else, it was defined as low. These cut-off values were determined as follows (Supplementary Table 2). First, patients who received the original dose would have a treatment delay of ≥7 days, which would result in the initial RDI being less than 70%. Second, if each agent was used at the -1 level (L-OHP, 65 mg/m^2^; CPT-11, 150 mg/m^2^; 5-FU bolus, omitted; 5-FU ci, 1800 mg/m^2^) and treatment delay was unnecessary in the first two cycles, the initial RDIs for L-OHP, CPT-11, 5-FU bolus, and 5-FU ci. in the first two cycles would be 76.5%, 83.3%, 0%, and 75.0%, respectively. Considering these values, we finally set the cut-off values to ensure that the observed data were exactly split accordingly (Figure 1).

The median OS and PFS and the corresponding 95% confidence intervals (CIs) were determined using the Kaplan–Meier method. The influence of the RDI of each agent on OS or PFS was evaluated using a log-rank test or multivariate Cox proportional hazard model to adjust for the patients’ background characteristics. Similar to the association between RDIs and patients’ backgrounds, the association between RDIs and objective responses was also evaluated using a multivariate logistic model. In all analyses, covariates were considered statistically significant if *p* was <0.05 for the null hypothesis of no effect.

## SUPPLEMENTARY MATERIALS FIGURES AND TABLES


