# Supplementary material for: Influence of initial dose intensity on efficacy of FOLFIRINOX in patients with advanced pancreatic cancer
Source: Oncotarget. 2019 Mar 5;10(19):1775–84. doi: 10.18632/oncotarget.26633 (PMC6442997; doi:10.18632/oncotarget.26633)
Supplement: Supplementary file 1 [file oncotarget-10-1775-s001.pdf]

Influence of initial dose intensity on efficacy of FOLFIRINOX in patients with advanced pancreatic cancer

SUPPLEMENTARY MATERIALS

Supplementary Table 1: Prognostic factors for overall survival (OS)

Variable	Hazard ratio	95% CI	*p*-value
L-OHP ≥ 70%	1.01	0.67–1.54	0.96
CPT-11 ≥ 75%	0.80	0.51–1.24	0.31
5-FU (bolus) > 0%	1.23	0.92–1.66	0.17
5-FU (duration) ≥ 80%	1.00	0.63–1.61	0.99
Sex	1.36	0.99–1.88	0.061
Age	1.00	0.74–1.36	0.98
Disease status			
Metastatic	1.42	0.95–2.13	0.086
Recurrence	1.83	1.14–2.93	0.012
History of therapy	1.76	1.29–2.42	<0.001
ECOG PS	1.63	1.18–2.24	0.003
G-CSF use	1.27	0.89–1.82	0.19
UGT1A1 (Ref: Wild)			
Single variant	0.85	0.64–1.15	0.29
Double variant	1.58	0.81–3.11	0.18
CA19-9*	1.60	1.20–2.15	0.001
Albumin*	1.08	0.75–1.56	0.67
CRP*	2.64	1.70–4.11	<0.0001

*continuous value.

Abbreviations: L-OHP, oxaliplatin; CPT-11, irinotecan, 5-FU, fluorouracil; ci, continuous infusion; ECOG PS, Eastern Cooperative Oncology Group Performance status; G-CSF, granulocyte-colony stimulating factor; CA19-9, carbohydrate antigen 19-9; CRP, C-reactive protein.

Supplementary Table 2: Comparison of patient's characteristics in high and low groups of relative dose intensity (RDI) of 5-FU bolus

Factor	RDI of 5-FU bolus, *n* (%)		*p*-value
	High, *n* = 180	Low, *n* = 179	
Sex, male	125 (69.4)	119 (66.5)	0.573*
Age ≧ 65	66 (36.7)	70 (39.1)	0.664*
Disease status			0.067*
Locally advanced	44 (24.4)	29 (16.2)	
Metastatic	96 (53.3)	116 (64.8)	
Recurrence	40 (22.2)	34 (19.0)	
History of prior chemotherapy	59 (32.8)	35 (19.6)	0.0056*
ECOG PS >0	57 (31.7)	45 (25.1)	0.1981*
UGT1A1			0.202*
Wild	110 (62.1)	91 (52.6)	
Single	60 (33.9)	74 (42.8)	
Double	7 (4.0)	8 (4.6)	
CA19-9, U/mL [median(range)]	977(0.4–260800)	1071(2–368500)	0.624**
Albumin, g/dL [median(range)]	3.9 (2.3–5.3)	3.9 (2.7–4.7)	0.648**
CRP, mg/dL [median(range)]	0.28 (0.00–11.2)	0.21 (0.00–12.4)	0.108**

*Fisher's exact test, **Mann–Whitney *U* test.
Abbreviations: ECOG PS, Eastern Cooperative Oncology Group performance status; CA19-9, carbohydrate antigen 19-9; CRP, C-reactive protein.

Supplementary Table 3: Typical model of treatment modification

	L-OHP			CPT-11		
Level 0	85 mg/m^2			180 mg/m^2		
Level -1	65 mg/m^2			150 mg/m^2		
First cycle	Level 0	Level 0	Level -1	Level 0	Level 0	Level -1
Second cycle	Level -1	Level 0	Level -1	Level -1	Level 0	Level -1
Treatment delay	+7 days	+7 days	0	+ 7days	+ 7days	0
RDI	58.8%	66.7%	76.5%	61.1%	67.7%	83.3%

Threshold of relative dose intensity (RDI) in this study was based on these models to divide the highest RDI treatment course (the right column in each agent) from the others.

	5-FU bolus			5-FU ci		
Level 0	240 mg/m^2			2400 mg/m^2		
Level -1	0 mg/m^2			1800 mg/m^2		
First cycle	Level 0	Level 0	Level -1	Level 0	Level -1	Level 0
Second cycle	Level -1	Level 0	Level -1	Level 0	Level -1	Level -1
Treatment delay	+7 days	+7 days	0	+ 7days	0	0
RDI	33.3%	66.7%	0%	66.7%	75.0%	87.5%

Abbreviations: L-OHP, oxaliplatin; CPT-11, irinotecan, 5-FU, fluorouracil; ci, continuous infusion; RDI, relative dose intensity.

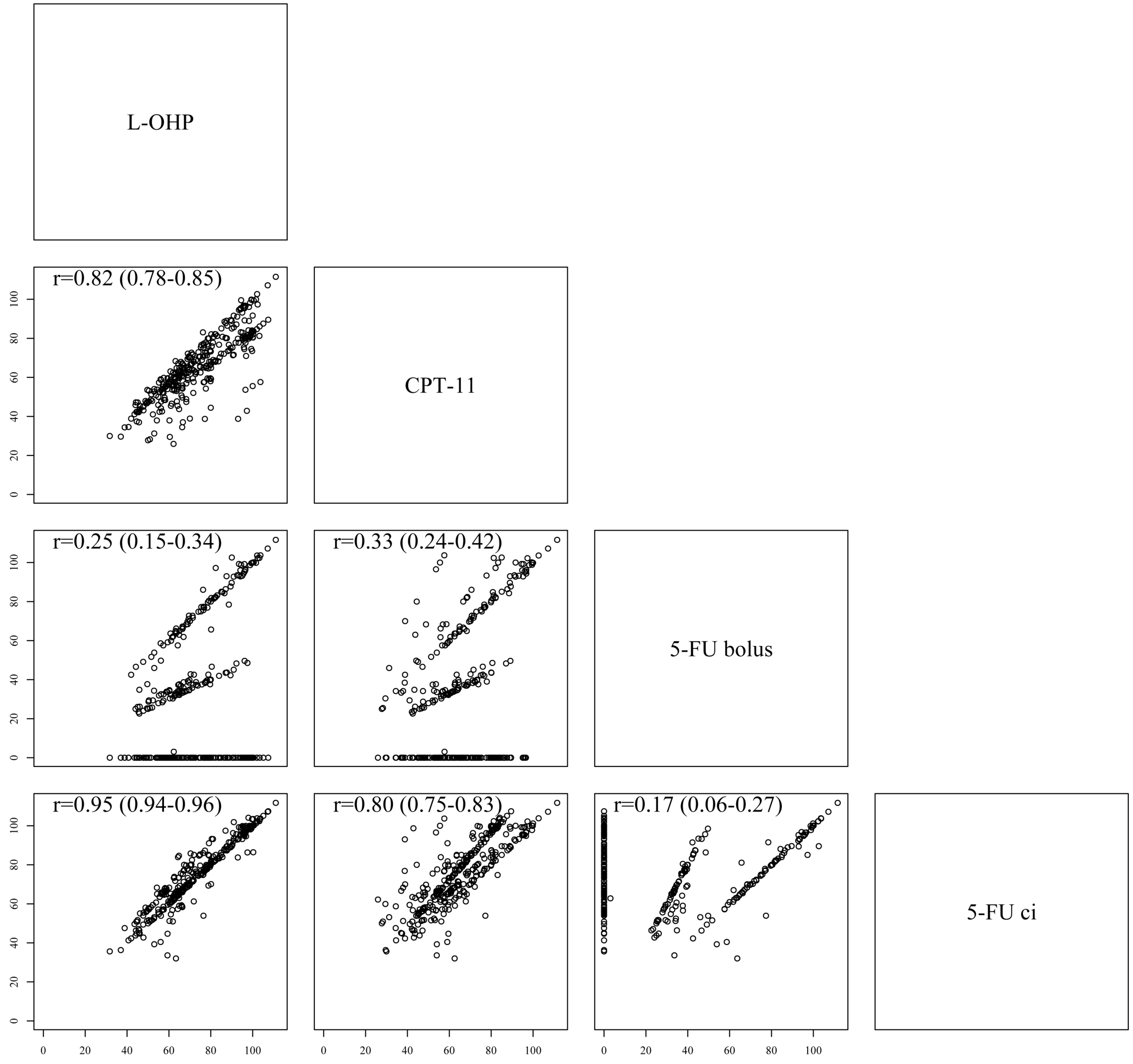

Supplementary Figure 1: Correlation of the relative dose intensities (RDIs) of each agent in FOLFIRINOX. The correlation coefficient (r) is described with the 95% confidence interval (CI) in each column. There were strong correlations in RDI between L-OHP and CPT-11, L-OHP and 5-FU ci, and CPT-11 and 5-FU ci. Abbreviations: L-OHP, oxaliplatin; CPT-11, irinotecan; 5-FU, fluorouracil; ci, continuous infusion.

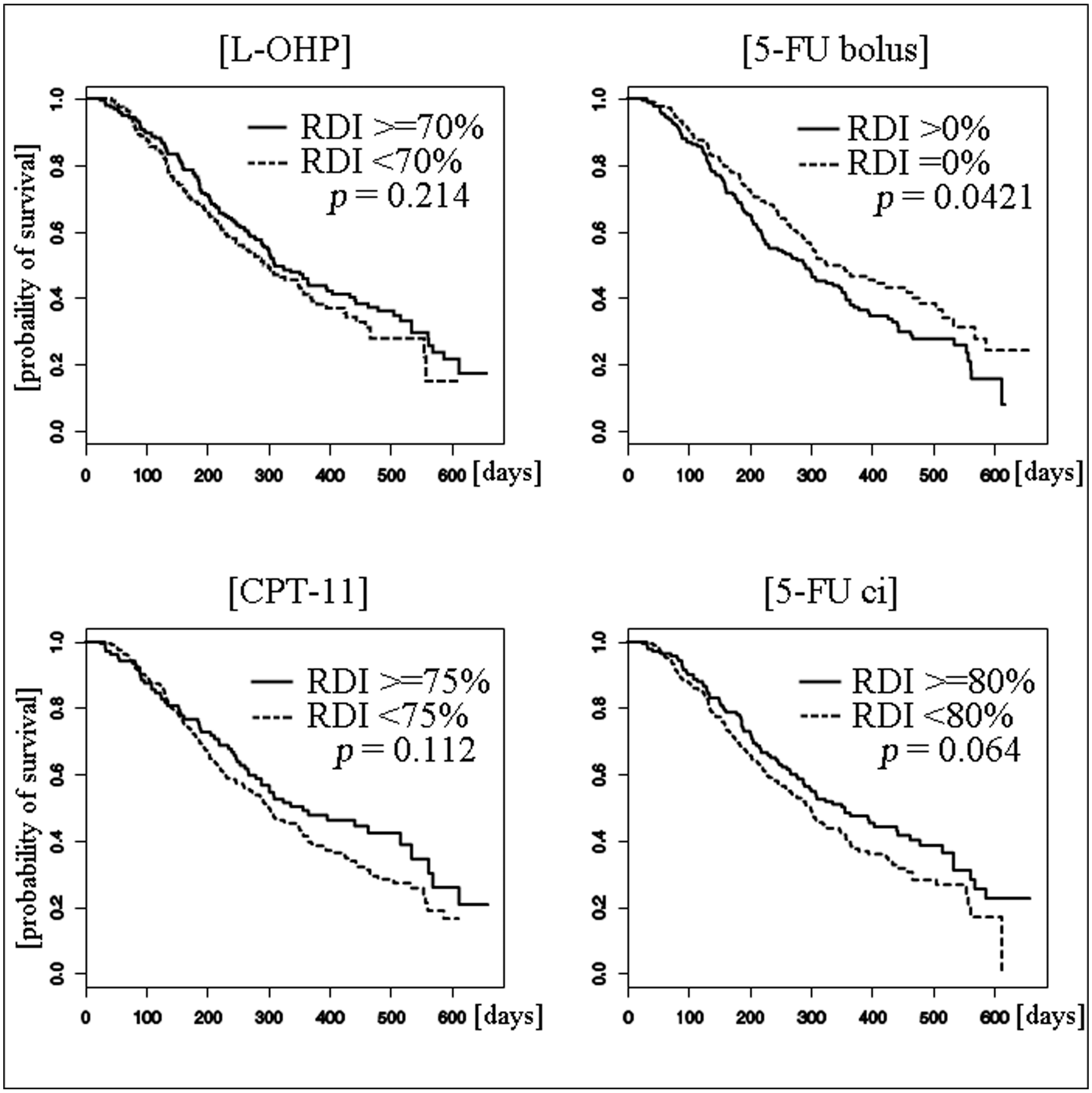

Supplementary Figure 2: Kaplan–Meier curves of overall survival (OS). Patients who received drugs at higher and lower relative dose intensities (RDIs) than the cut-off values are represented by solid and dotted lines, respectively. There was a statistical difference in overall survival between patients who received 5-FU bolus at higher and lower relative dose intensities (> 0% vs. 0%, $p = 0.042$); no such differences were observed for L-OHP (≥ 70% vs. < 70%), CPT-11 (≥ 75% vs. < 75%), and 5-FU ci (≥ 80% vs. < 80%, p = 0.21, 0.11, and 0.064, respectively). Abbreviations: L-OHP, oxaliplatin; CPT-11, irinotecan; ci, 5-FU, fluorouracil; continuous infusion.
